# A new species of the genus *Anteon* Jurine (Hymenoptera, Dryinidae) from Laos

**DOI:** 10.3897/zookeys.561.7417

**Published:** 2016-02-08

**Authors:** Massimo Olmi, Zai-fu Xu, Adalgisa Guglielmino, Stefano Speranza

**Affiliations:** 1 Tropical Entomology Research Center, Viterbo, Italy; 2 Department of Entomology, South China Agricultural University, Guangzhou, Guangdong, P.R. China; 3 Department of Agriculture and Forestry Sciences (DAFNE), University of Tuscia, Viterbo, Italy

**Keywords:** Taxonomy, Anteon holzschuhi, Oriental region, key, Houaphanh Province, Anteoninae

## Abstract

A new species of *Anteon* Jurine, 1807 is described from Laos, Houaphanh Province: *Anteon holzschuhi* **sp. n**. Morphologically the new species is similar to *Anteon semipolitum* Olmi, 2008, but it is distinguished by the sculpture of the face partly reticulate rugose and partly with deep punctures similar to areolae; in *Anteon semipolitum* the face is completely punctate and unsculptured among punctures. In addition, in the new species the distance from the outer edge of a lateral ocellus to the compound eye (OOL) is about 3.3 times as long as the distance between the inner edges of a lateral ocellus and the median ocellus (OL); in *Anteon semipolitum* OOL is less than twice as long as OL. Published identification keys to the Oriental species of *Anteon* are modified to include the new species.

## Introduction

Dryinidae (Hymenoptera, Chrysidoidea) are parasitoids of leafhoppers, planthoppers and treehoppers (Hemiptera, Auchenorrhyncha) (Carcupino et al. 1998; Guglielmino and Bückle 2003, 2010; Guglielmino et al. 2006, 2013, 2015; Guglielmino and Virla 1998). *Anteon* Jurine, 1807 is a genus that is present in all zoogeographical regions (Olmi 1984; Xu et al. 2013; Olmi and Virla 2014; Olmi and Xu 2015). In total 423 species have been described from all continents (Olmi and Xu 2015) and the genus was revised at the world level by Olmi (1984, 1991) and more recently in the Oriental, Neotropical and Eastern Palaearctic regions by Xu et al. (2013), Olmi and Virla (2014) and Olmi and Xu (2015) respectively.

The species of *Anteon* inhabiting the Oriental region were studied by Xu et al. (2013). More recently, Guglielmino and Olmi (2013) and Olmi et al. (2015) described further new species respectively from Indonesia (*Anteon seramense* Guglielmino & Olmi) and Thailand (*Anteon huettingeri* Olmi, Xu & Guglielmino). In total, 150 *Anteon* species have been described from the Oriental region (Xu et al. 2013; Guglielmino and Olmi 2013; Olmi et al. 2015).

*Anteon* species are parasitoids of leafhoppers belonging to the Cicadellidae (Guglielmino et al. 2013). As in almost all dryinids, females of *Anteon* have a chelate protarsus. Chelae are used to capture and restrain the host during oviposition and host-feeding (Olmi 1984, 1994).

In 2015 we examined additional specimens of *Anteon* from Laos and discovered a new species described in this paper.

## Material and methods

The descriptions follow the terminology used by Olmi (1984) and Xu et al. (2013). The measurements reported are relative, except for the total length (head to abdominal tip, without antennae), which is expressed in millimetres. The following abbreviations are used in the descriptions: POL is the distance between the inner edges of the two lateral ocelli; OL is the distance between the inner edges of a lateral ocellus and the median ocellus; OOL is the distance from the outer edge of a lateral ocellus to the compound eye; OPL is the distance from the posterior edge of a lateral ocellus to the occipital carina; TL is the distance from the posterior edge of an eye to the occipital carina.

The types of all Oriental species of *Anteon* have been previously examined by the authors.

The type specimen described in this paper is deposited in the collection of the Oberösterreichisches Landesmuseum, Linz, Austria (OLL).

The description of the new species is based on the study of a single specimen. The authors are aware that descriptions of new taxa should normally be based on more individuals. However, Dryinidae are so rare that it is uncommon to collect more than one specimen of each species. In addition, on the basis of the experience and knowledge of the authors, the new species is sufficiently delimited by unique characters to justify its description.

## Results

### Genus Anteon

Taxon classificationAnimaliaHymenopteraDryinidae

Jurine, 1807

Anteon Jurine, 1807: 302. Type species: *Anteon jurineanum* Latreille, 1809, by subsequent monotypy.

#### Diagnosis.

Female: Fully winged; rarely brachypterous; occipital carina complete; palpal formula 6/3; antenna without rhinaria; forewing with three cells enclosed by pigmented veins (costal, median and submedian); forewing with stigmal vein and pterostigma; distal part of stigmal vein much shorter than proximal part, occasionally slightly shorter, as long as, or longer than proximal part; propodeum usually with transverse keel between dorsal and posterior surface; protarsus chelate; inner side of enlarged claw with proximal prominence bearing one long bristle; tibial spurs 1/1/2. Male: Fully winged; rarely brachypterous; occipital carina complete; vertex of head usually without two oblique keels connecting posterior ocelli to occipital carina; palpal formula 6/3; forewing with three cells enclosed by pigmented veins (costal, median and submedian); forewing with stigmal vein and pterostigma; distal part of stigmal vein much shorter than proximal part, occasionally slightly shorter, as long as, or longer than proximal part; pterostigma less than four times as long as broad; propodeum usually with transverse keel between dorsal and posterior surface; paramere usually without inner branch wrapping penis; tibial spurs 1/1/2.

### Anteon holzschuhi

Taxon classificationAnimaliaHymenopteraDryinidae

Olmi, Xu, Guglielmino & Speranza sp. n.

http://zoobank.org/80257611-458A-45B3-B633-292B862C17A3

#### Diagnosis.

**Figure 1.**
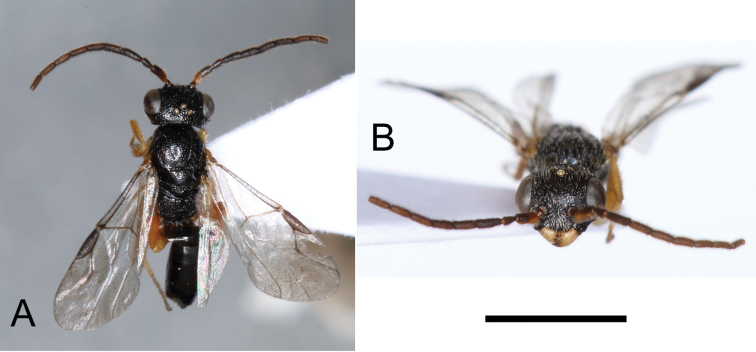
*Anteon holzschuhi* Olmi, Xu, Guglielmino & Speranza, sp. n., holotype: **A** habitus in dorsal view **B** head in frontal view. Scale bar: 2.77 mm (**A**), 2.20 mm (**B**).

**Figure 2.**
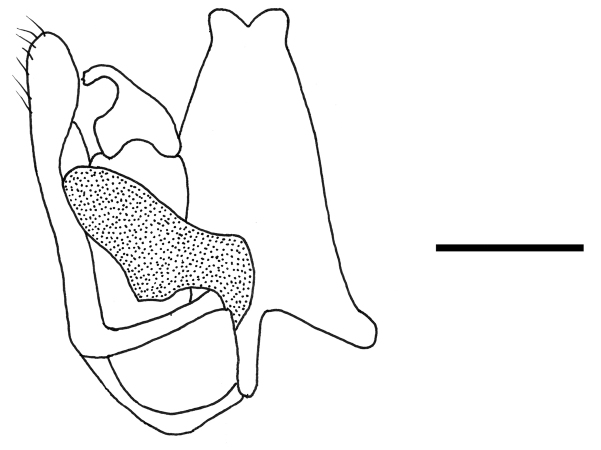
*Anteon holzschuhi* Olmi, Xu, Guglielmino & Speranza, sp. n., holotype: male genitalia (right half removed). Scale bar = 0.26 mm.

Male with antenna filiform; face partly reticulate rugose and partly sculptured by deep punctures similar to areolae (Fig. 1B); OOL about 3.3 times as long as OL (Fig. 1A); notauli reaching about 0.8 length of scutum (Fig. 1A); posterior surface of propodeum with two complete longitudinal keels and median area unsculptured; distal part of stigmal vein much shorter than proximal part; paramere about as long as penis, without papillae on inner side, without distal inner process (Fig. 2); distivolsella not provided with two lateral processes (Fig. 2).

#### Description.

**Male.** Fully winged (Fig. 1A). Length 4.5 mm. Head black, except mandible testaceous. Antenna brown-testaceous, except proximal half of segment 1 testaceous. Mesosoma black. Metasoma brown. Legs testaceous, except metacoxa basally brown. Antenna filiform. Antennal segments in following proportions: 17:10:16:15:14:14:14:14:13 (segment 10 missing in holotype). Head (Fig. 1A, B) shiny. Face partly rugose and partly strongly punctate, with deep punctures similar to areolae, unsculptured among punctures. Vertex and temple with deep punctures similar to areolae, unsculptured among punctures. Frontal line complete. Vertex with POL = 7; OL = 3; OOL = 10; OPL = 7; TL = 7; greatest breadth of posterior ocelli shorter than OPL (5:7). Occipital carina complete. Pronotum short and strongly punctate. Scutum and scutellum shiny, punctate, unsculptured among punctures. Notauli incomplete, reaching approximately 0.8 x length of scutum (Fig. 1A). Metanotum shiny, unsculptured. Propodeum with strong transverse keel between dorsal and posterior surface. Dorsal surface of propodeum reticulate rugose. Posterior surface of propodeum with two complete longitudinal keels, median area unsculptured, and lateral areas rugose. Forewing hyaline, without dark transverse bands. Distal part of stigmal vein much shorter than proximal part (7:16). Paramere (Fig. 2) about as long as penis, without distal inner pointed process and papillae, with long and broad dorsal proximal membranous process. Tibial spurs 1/1/2.

**Female.** Unknown.

#### Material examined.

**Holotype**: male, Laos, Houaphanh Province, Phou Pan, Ort Ban Saleui environs, 20°13.30'N 103°59.26'E, 1350–1900 m, 6–11.iv.2014, C. Holzschuh and locals leg. (OLL).

#### Distribution.

Laos.

#### Hosts.

Unknown.

#### Etymology.

The species is named after the collector, Mr Carolus Holzschuh (Villach, Austria).

#### Remarks.

The new species is similar to *Anteon semipolitum* Olmi, 2008, by having the antenna filiform, notauli reaching about 0.8 × length of scutum (Fig. 1A), posterior surface of the propodeum with two complete longitudinal keels and unsculptured median area, distal part of stigmal vein much shorter than proximal part, paramere about as long as penis, without papillae on inner side, without distal inner process (Fig. 2) and distivolsella not provided with two lateral processes (Fig. 2). The main difference between the two species is in the facial sculpture (face partly reticulate rugose and partly sculptured by deep punctures similar to areolae in *Anteon holzschuhi* (Fig. 1B); face punctate and unsculptured among punctures in *Anteon semipolitum*). In addition, OOL is about three times as long as OL in *Anteon holzschuhi*, less than twice in *Anteon semipolitum*. In the key to the males of Oriental *Anteon* published by Xu et al. (2013), the new species can be included by replacing couplet 43 as follows:

**Table:** 

43	Head partly or totally reticulate rugose (Fig. 1A, B)	**43**’
–	Head completely punctate and unsculptured among punctures	**44**
43’	Paramere with many papillae along inner side (Plate 38H in Xu et al. 2013)	***Anteon papillum* Xu, He & Olmi**
–	Paramere without papillae on inner side (Fig. 2; plate 42F in Xu et al. 2013)	**43**’’
43’’	Face punctate and unsculptured among punctures; OOL less than twice as long as OL	***Anteon semipolitum* Olmi**
–	Face partly reticulate rugose and partly sculptured by deep punctures similar to areolae (Fig. 1B); OOL about 3.3 times as long as OL (Fig. 1A)	***Anteon holzschuhi* Olmi, Xu, Guglielmino & Speranza, sp. n.**

## Conclusion

Mita and Okajima (2011), Xu et al. (2013) and Olmi et al. (2015) recorded from Laos 41 species of Dryinidae belonging to the following subfamilies and genera: Aphelopinae: *Aphelopus* Dalman, 1823 (five species); Anteoninae: *Anteon* Jurine, 1807 (nine species), *Deinodryinus* Perkins, 1907 (one species); Bocchinae: *Bocchus* Ashmead, 1893 (four species); Dryininae: *Dryinus* Latreille, 1804 (16 species); Gonatopodinae: *Neodryinus* Perkins, 1905 (four species), *Echthrodelphax* Perkins, 1903 (two species). With the description of the above new species the number of species now known in Laos is 42. No hosts of Laotian Dryinidae are known.

In comparison with the 77 species listed in the Chinese province of Guangdong (total area: 177900 km^2^) (Xu et al. 2012c), the dryinid fauna of Laos (total area: 237800 km^2^) is poorly known. Some common genera such as *Gonatopus* Ljungh, 1810 (no species listed in Laos) are clearly understudied. Further evidence of this is the fact that on the small island of Hainan (total area: 33210 km^2^; 1/7 that of Laos) 56 species of Dryinidae are recorded (Xu et al. 2011a, 2011c). In contrast, in the more northern Chinese provinces of Shaanxi (total area: 205800 km^2^) and Hunan (total area: 210000 km^2^) only 36 (Xu et al. 2012a) and 17 (Xu et al. 2011b, 2012b, 2013) dryinid species have been recorded, respectively.

## Supplementary Material

XML Treatment for Anteon

XML Treatment for Anteon holzschuhi
